# The heuristics of nurse responsiveness to critical patient monitor and ventilator alarms in a private room neonatal intensive care unit

**DOI:** 10.1371/journal.pone.0184567

**Published:** 2017-10-05

**Authors:** Rohan Joshi, Heidi van de Mortel, Loe Feijs, Peter Andriessen, Carola van Pul

**Affiliations:** 1 Department of Industrial Design, Eindhoven University of Technology, Eindhoven, The Netherlands; 2 Department of Clinical Physics, Máxima Medical Center, Veldhoven, The Netherlands; 3 Department of Neonatology, Máxima Medical Center, Veldhoven, The Netherlands; 4 Department of Pediatrics, Maastricht University Medical Center, Faculty of Health, Medicine and Life Sciences, School for Mental Health and Neuroscience, Maastricht, The Netherlands; 5 Department of Applied Physics, Eindhoven University of Technology, Eindhoven, The Netherlands; Waseda University, JAPAN

## Abstract

**Aim:**

Alarm fatigue is a well-recognized patient safety concern in intensive care settings. Decreased nurse responsiveness and slow response times to alarms are the potentially dangerous consequences of alarm fatigue. The aim of this study was to determine the factors that modulate nurse responsiveness to critical patient monitor and ventilator alarms in the context of a private room neonatal intensive care setting.

**Methods:**

The study design comprised of both a questionnaire and video monitoring of nurse-responsiveness to critical alarms. The Likert scale questionnaire, comprising of 50 questions across thematic clusters (critical alarms, yellow alarms, perception, design, nursing action, and context) was administered to 56 nurses (90% response rate). Nearly 6000 critical alarms were recorded from 10 infants in approximately 2400 hours of video monitoring. Logistic regression was used to identify patient and alarm-level factors that modulate nurse-responsiveness to critical alarms, with a response being defined as a nurse entering the patient’s room within the 90s of the alarm being generated.

**Results:**

Based on the questionnaire, the majority of nurses found critical alarms to be clinically relevant even though the alarms did not always mandate clinical action. Based on video observations, for a median of 34% (IQR, 20–52) of critical alarms, the nurse was already present in the room. For the remaining alarms, the response rate within 90s was 26%. The median response time was 55s (IQR, 37-70s). Desaturation alarms were the most prevalent and accounted for more than 50% of all alarms. The odds of responding to bradycardia alarms, compared to desaturation alarms, were 1.47 (95% CI = 1.21–1.78; <0.001) while that of responding to a ventilator alarm was lower at 0.35 (95% CI = 0.27–0.46; p <0.001). For every 20s increase in the duration of an alarm, the odds of responding to the alarm (within 90s) increased to 1.15 (95% CI = 1.1–1.2; p <0.001). The random effect per infant improved the fit of the model to the data with the response times being slower for infants suffering from chronic illnesses while being faster for infants who were clinically unstable.

**Discussion:**

Even though nurses respond to only a fraction of all critical alarms, they consider the vast majority of critical and yellow alarms as useful and relevant. When notified of a critical alarm, they seek waveform information and employ heuristics in determining whether or not to respond to the alarm.

**Conclusion:**

Amongst other factors, the category and duration of critical alarms along with the clinical status of the patient determine nurse-responsiveness to alarms.

## Introduction

Alarm fatigue is a well-recognized patient safety concern in intensive care settings [1–6]. It arises because alarm pressure is high, the clinical significance of alarms is limited, and only a small fraction of all alarms redirects the attention of the nurse to the patient, with fewer still leading to clinical action [7]. The continually increasing number of physiological parameters that can be monitored, alongside a trend towards private patient room care and the accompanying decrease in the line of sight compounds the challenge of patient monitoring, and by association alarm fatigue. Decreased responsiveness and slow response times to alarms are the potentially dangerous consequences of alarm fatigue.

In a previous study in a neonatal intensive care unit (NICU) with private rooms, we have shown that, while challenging, safe patient monitoring is feasible [8]. That study ascertained safety by the low frequency of repeated desaturation and bradycardia alarms. These ‘repeat’ alarms arose when the original alarm condition did not subside within 45s, and the nurse did not respond by silencing or pausing the alarm on the patient monitor or the handheld devices within this period. In the neonatal context, fast response times are particularly important because of the risk of neurological morbidity. For instance, prolonged episodes of hypoxemia (>60s) among infants born at a gestational age of 27 weeks or lower has been associated with adverse 18-month outcomes including mortality, motor impairment, cognitive or language delay, severe hearing loss and bilateral blindness [9]. Even if individual desaturation alarms are not long-lasting, the fact that they often occur in clusters suggests that the physiological mechanisms for increasing oxygenation are slow [10]. In this light, adequate nursing response times are essential to enable intervention offsetting potentially dangerous physiological derangements.

Research has shown that nurses do not respond to all alarms but, in fact, integrate information from multiple sources in a heuristic fashion in their decision making [2,11,12]. Within the context of a private room, with limited visual oversight, increasing patient surveillance via technological means and the potential need to cover long distances to reach the bedside, the complexity associated with addressing alarm fatigue acquires new dimensions [13]. Surprisingly, limited research has been carried out on alarm management, the clinical relevance of alarms and the factors that modulate nurse responsiveness, especially for private room settings [14]. Therefore, the goal of this study was to, (i) survey nurses on alarms across broad themes in the context of a private room NICU and (ii) to contrast these subjective findings against an objective video-annotated measure of nurse responsiveness to critical alarms.

## Materials and methods

### Clinical setting and alarm architecture

This prospectively designed observational study was conducted in the NICU of Máxima Medical Centre, the Netherlands (level III; tertiary care NICU; private-room design) between February 2017 and June 2017 during which period the occupancy rate was 75%. The NICU is of private room design with 18 beds and is divided into two units. The architectural layout of each unit consists of a central nursing station at the head of a long corridor, with private rooms on both sides. All critical (red) and alerting (yellow) alarms from patient monitors and ventilators were automatically logged in a data warehouse (PIIC iX, Data Warehouse Connect, Philips Medical Systems, Andover, MA). These alarms were also displayed, in real time, on patient monitors (Philips IntelliVue MX 800, Germany), the inter-bed communication system, and the central post. Also, critical alarms were transmitted to handheld devices carried by the nurses. It should be noted that the terms red and yellow alarms are specific to Philips monitors and that other manufacturers may use different terminology to refer to critical and alerting alarms. With regard to the nurse patient ratio, typically it is 1:2, although it might decrease for short durations of time when some nurses are having a break.

Oxygen saturation was monitored using disposable pulse oximetry sensors (RD SET Sensors, Massimo SET^®^) with a signal averaging setting of 10s and alarms being generated after a 10s delay if the oxygen saturation dropped to and remained below predefined thresholds (80% for critical alarms and 85% for yellow alarms). Bradycardia alarms were generated by measuring the heart rate using a three-lead ECG sensor (3M^™^ Critical Dot^™^ or Ambu^®^ BlueSensor) with heart rate calculated as the average of the 12 most recent R-R intervals or the four most recent R-R intervals if the heart rate was less than 80 bpm. Critical bradycardia alarms were generated as soon as the heart rate fell below 80 bpm while yellow alarms were generated if the heart rate fell below 100 bpm. A detailed discussion of the alarm chain and architecture is provided in previous publications [8,10]. With regard to critical ventilator alarms, the three categories of alarms with physiological implications included the high peak inspiratory pressure alarm, disconnection of the ventilator circuit and problems with the endotracheal tube.

### Questionnaire for nurses

A questionnaire was distributed among all NICU nurses (n = 62) to collect data on the perception of alarms and the determinants of alarm fatigue in a private room context. All nurses who participated in the study had similar educational qualification including a bachelor’s degree in nursing and specialized training to provide neonatal intensive care. The questionnaire consisted of 50 questions based on a five-point Likert scale (except two questions that used a 3-point scale to collect nursing metadata). These questions were broadly divided across six thematic clusters—critical alarms (red alarms), yellow alarms, perception, design, nursing action, and context, but were administered to participants in an interspersed fashion. Thirty-five questions were collated from existing questionnaires such as the validated questionnaire of Torabizadeh et al. that was used on 102 ICU nurses and a questionnaire from the healthcare technology foundation which, while not validated, had more than 5600 respondents [15,16]. Thirteen additional questions, specifically tailored to a private room context were added to this group of questions. This questionnaire was piloted and reiterated based on feedback from five nurses with past NICU experience, but who no longer worked in the NICU. The final printed questionnaires were placed in envelopes and distributed amongst nurses with instructions to fill in the questionnaire in a private setting to avoid potential bias from colleagues. The questionnaire was administered in Dutch, the native language of the participations but the results have been translated into English for the purpose of this study. Notably, no personal information was collected in the questionnaire, and thus the results were, by default, anonymized. Information on the age and work experience of the nurses were obtained from the records of the human resources department.

### Video monitoring of patient rooms

We defined critical alarms to be clinically relevant if the nurse responded to the alarm by entering the patient’s room within the 90s of the alarm being generated. In other words, these alarms generated sufficient concern in the nurse to stop any ongoing task and walk into the patient’s room. It is noteworthy that, irrespective of location in the NICU, all critical alarms are received on the handheld devices carried by nurses. We identified that the nurse entered the patient room by using video monitoring. We did so by programming a small computer (Raspberry Pi 3, Raspberry Pi Foundation, UK) that was interfaced to an 8-megapixel infrared camera (2.3 x 2.5 x 0.9 cm, Pi NoIR) to take an image every 5s. The image was saved with the date-time as the file name to enable easy chronological ordering of the images. The camera was affixed to the top of the patient monitor, facing the door to the patient room and was connected to the computer using a ribbon cable. The Raspberry Pi computer was mounted at the back of the patient monitor, away from sight.

Based on the definition of nurse-responsiveness employed in this study, we chose to acquire images every 5s since that provided sufficient temporal resolution and vastly reduced the data collected. Moreover, chronological ordering of all images facilitated and speeded up the manual inspection of those images that corresponded to the short windows of time around the occurrence of alarms. This inspection of images was carried out by co-author and senior nurse HvdM.

### Patient selection

Ten infants were enrolled in this study with the intention of video-monitoring each of them for 7–10 days. These infants were selected by availability and the likelihood of producing an abundance of critical alarms. During the study, two infants were diagnosed with late-onset sepsis, one was diagnosed with necrotizing enterocolitis (NEC; who later died), one was diagnosed with grade II intraventricular hemorrhage (IVH), two suffered from bronchopulmonary dysplasia (BPD) and one suffered from respiratory distress syndrome (RDS) for at least one of the study days. One infant was on invasive ventilation for one or more days of the study.

Written informed consent was obtained from the parents of infants for the use of video monitoring. Since the remainder of the data that was used corresponded to routine patient monitoring, a waiver was provided by the medical ethical committee in accordance with the Dutch law on medical research with humans (WMO). With regard to the nurses, general informed consent for video monitoring was obtained from the nursing management and was ratified by the medical ethical committee. All nurses were informed about video monitoring via a newsletter with the possibility to decline participation. None of the nurses declined participation.

### Data analysis

The findings of the questionnaire, regarding percentage agreement with the different categories of the Likert scale, were graphically presented in color-coded bar charts while data from video observations were summarized with median and interquartile ranges.

The responsiveness of the nurses to critical alarms within 90s was modeled using logistic regression with fixed and random effects. The predictor variables included birth weight, post-menstrual age, postnatal age, the category of alarm, duration of alarms and random effects per infant. The regression coefficients were calculated using maximum likelihood estimates. The category of alarms was dummy coded using the ‘reference’ method with ‘desaturation’ alarm as the reference. The fit of the regression model to the data was tested using the F-test while the likelihood ratio test was used to check the goodness of fit of the model after incorporating a random effect.

For the overall model, the statistical significance was reported. For all statistically significant effects, the odds ratio (OR) and their 95% CI were reported. Given the fact that the logistic function has a variance of π^2^/3, the contribution of the random effect to the overall variance (π^2^/3 + sum of variance contributions of each random effect term) of the model was calculated. All data were analyzed using Matlab R2015b (MathWorks, USA) and R (R Foundation for Statistical Computing, Vienna, Austria). The Raspberry Pi was programmed using Python (Python Software Foundation). Statistical significance was assumed if the P-value was < 0.01.

## Results

In total, 56 nurses completed the questionnaire corresponding to a response rate of 90%. All but one respondent was female. Nearly 70% of the respondents were between 35–55 years of age, and 75% of the respondents had more than ten years of work experience in a NICU. 42% of the respondents worked less than 29 hours a week while the remainder worked between 29–36 hours weekly.

**Fig 1:**
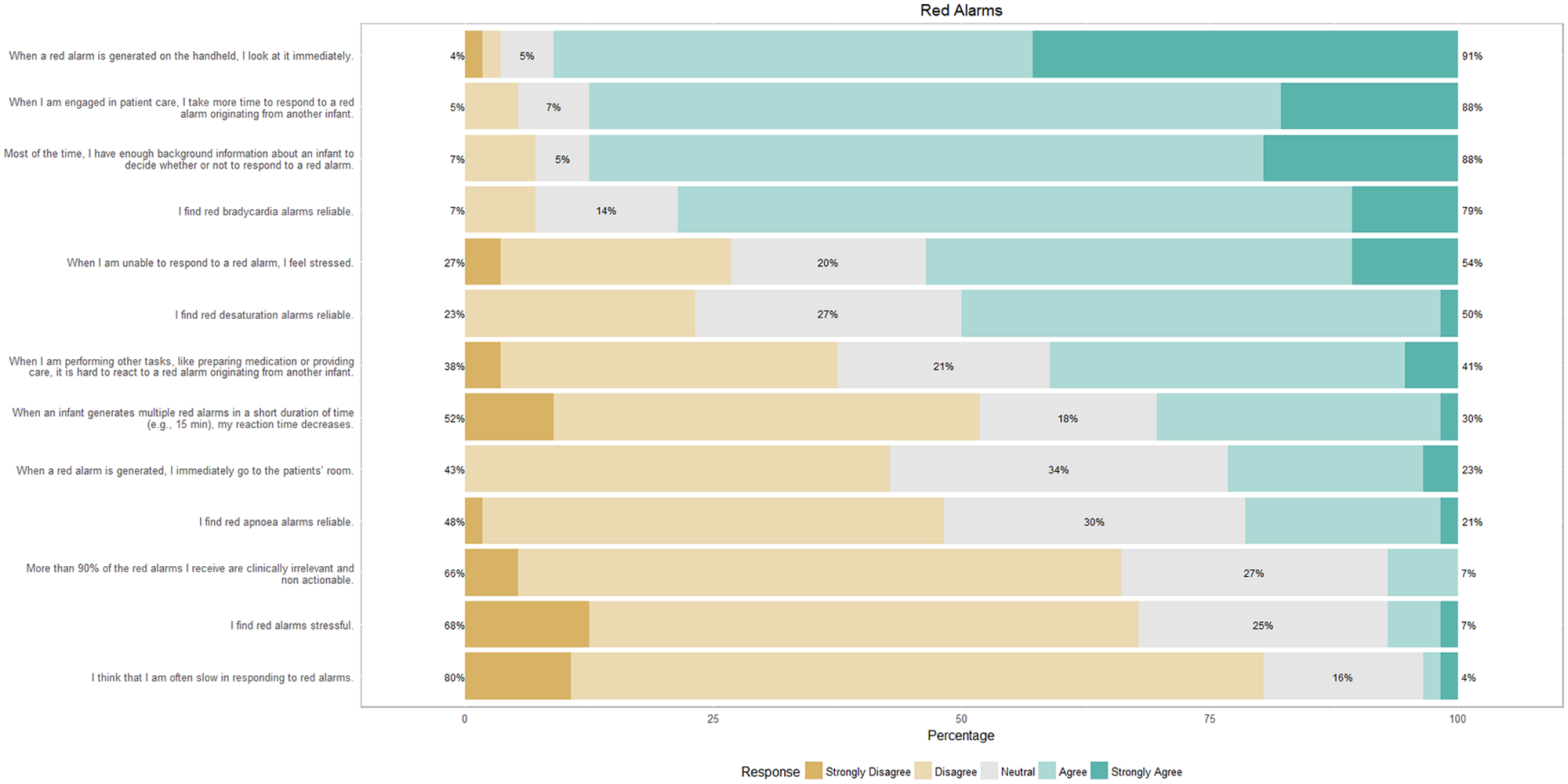
Response of nurses on a survey on critical (red) alarms.

**Fig 2:**
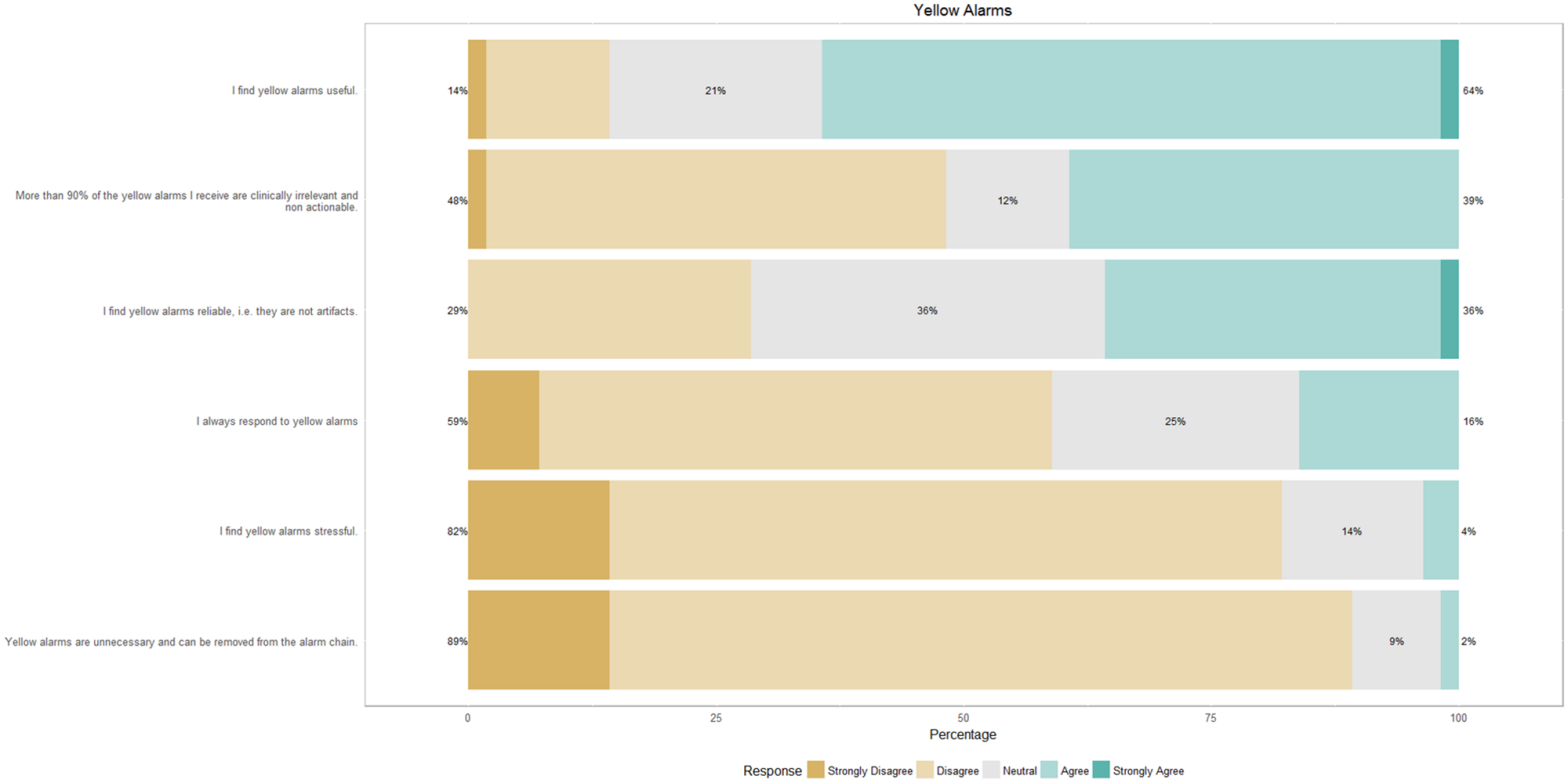
Response of nurses on a survey on yellow alarms.

Fig 1 shows the response of nurses to questions on critical alarms. When a critical alarm is generated, nurses immediately look at their handheld devices and use additional sources of information (e.g., the infant’s background) to determine whether or not to respond to the alarm. While they believe that the majority of critical alarms are clinically relevant, only 23% respond by immediately going to the patient’s room. About yellow alarms (Fig 2), nurses find these alarms to be useful but of limited clinical relevance. Typically, they do not respond to yellow alarms but would like yellow alarms to remain in the alarm chain.

**Fig 3:**
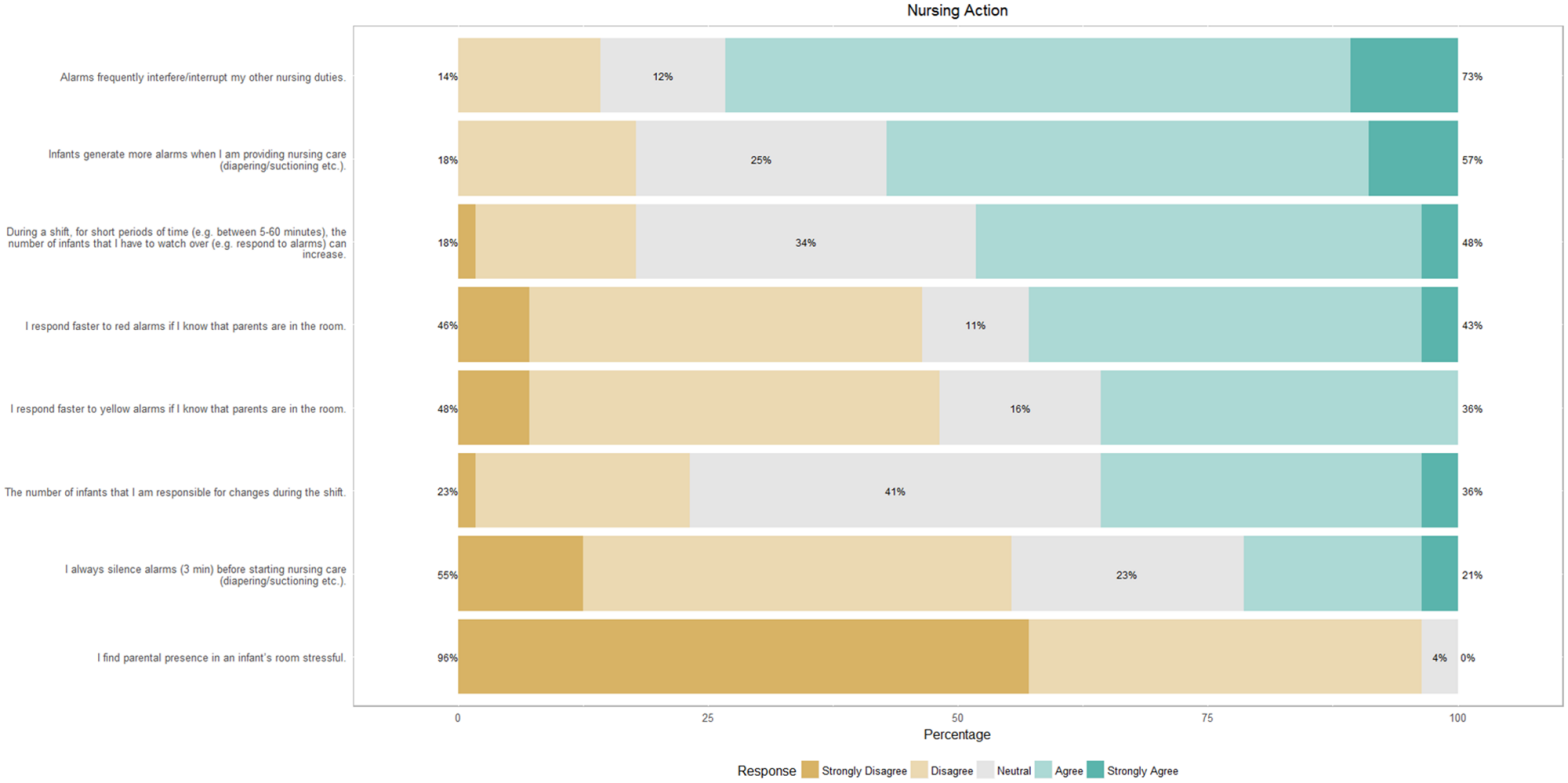
Response of nurses on a survey on nursing action and their association with alarms.

Fig 3 shows the response regarding nursing action. In particular, nurses find that alarms interfere with their other nursing duties and that infants generate more alarms when nursing care is provided. They rarely silence alarms before nursing care and don’t find parental presence in the room to be stressful, although a significant number of nurses tend to respond faster to alarms if parents are in the room.

**Fig 4:**
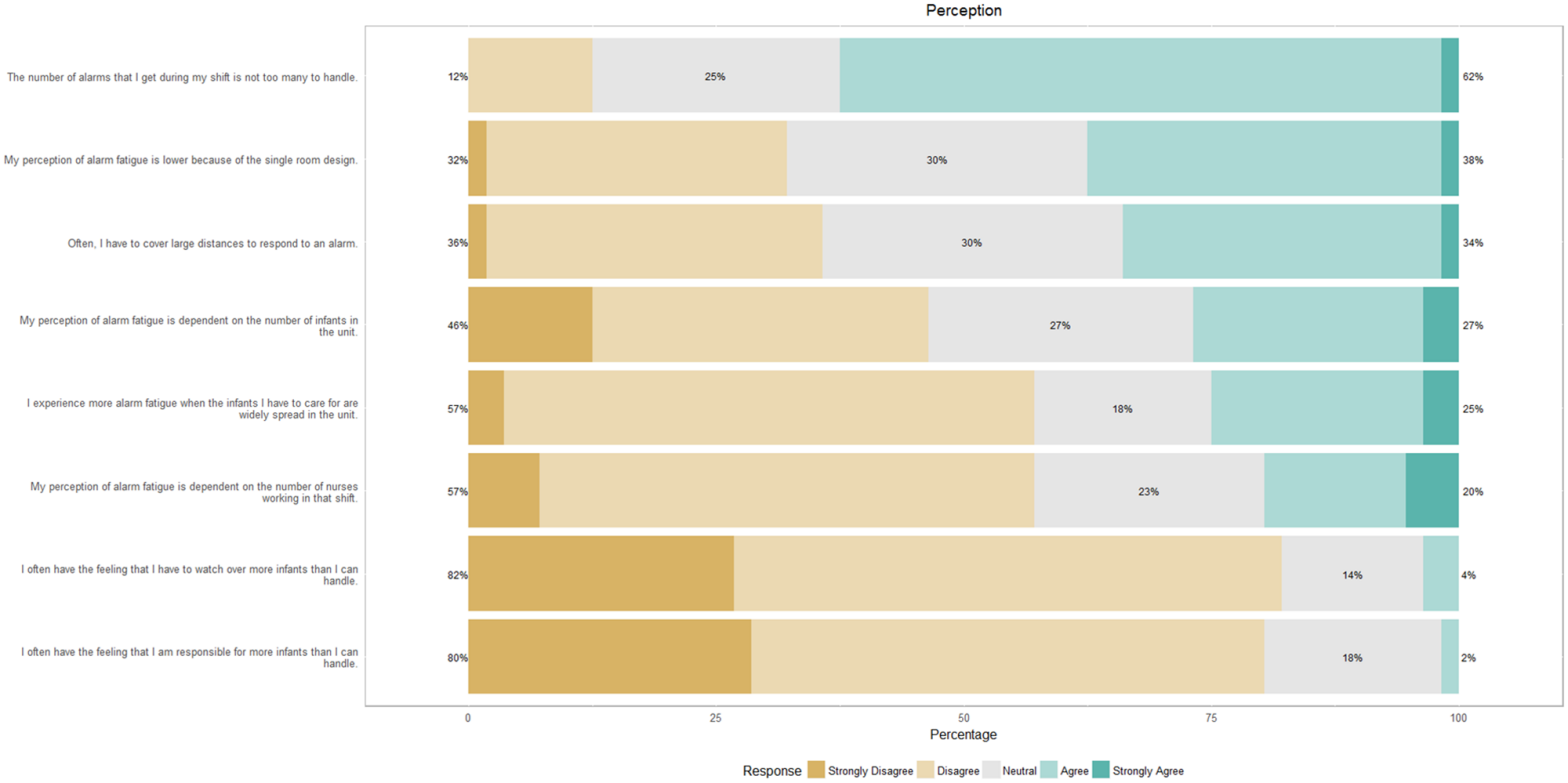
Nursing perceptions on alarms.

Fig 4 shows that nurses do not perceive to receive more alarms than they can handle in a shift. They are neutral about the contribution of a private room design in lowering alarm fatigue. The number of infants in the unit, the number of infants they have to care for and the number of nurses working the shift does not color their perception of alarm fatigue.

**Fig 5:**
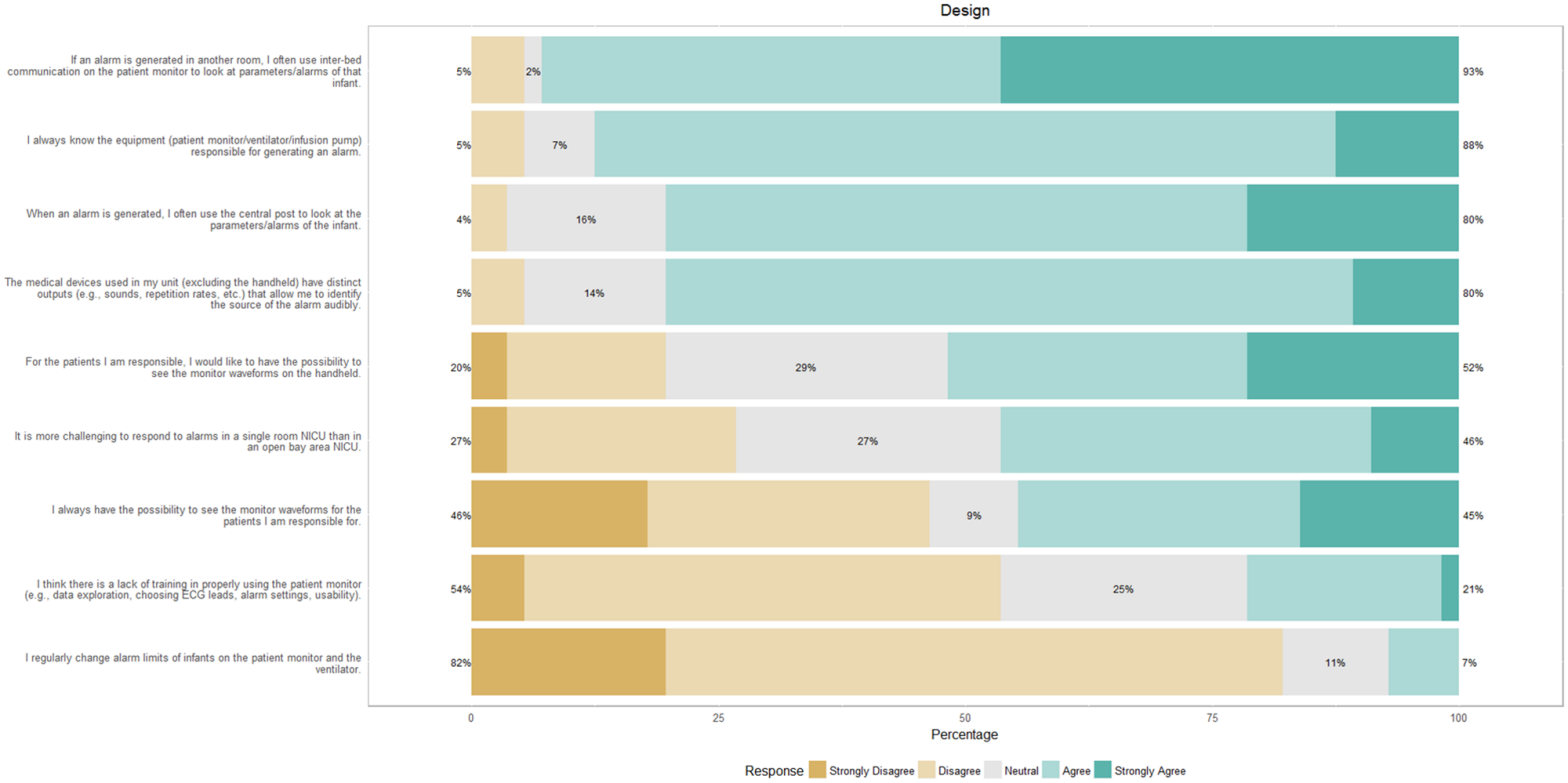
Response of nurses on how design affects alarms.

Fig 5 shows that nurses are aware of which equipment is generating the alarm and can audibly identify the source of the alarm. The inter-bed communication and the central post are almost always used when addressing alarms, and more than half the nurses would like to be able to see the waveforms on their handhelds.

**Fig 6:**
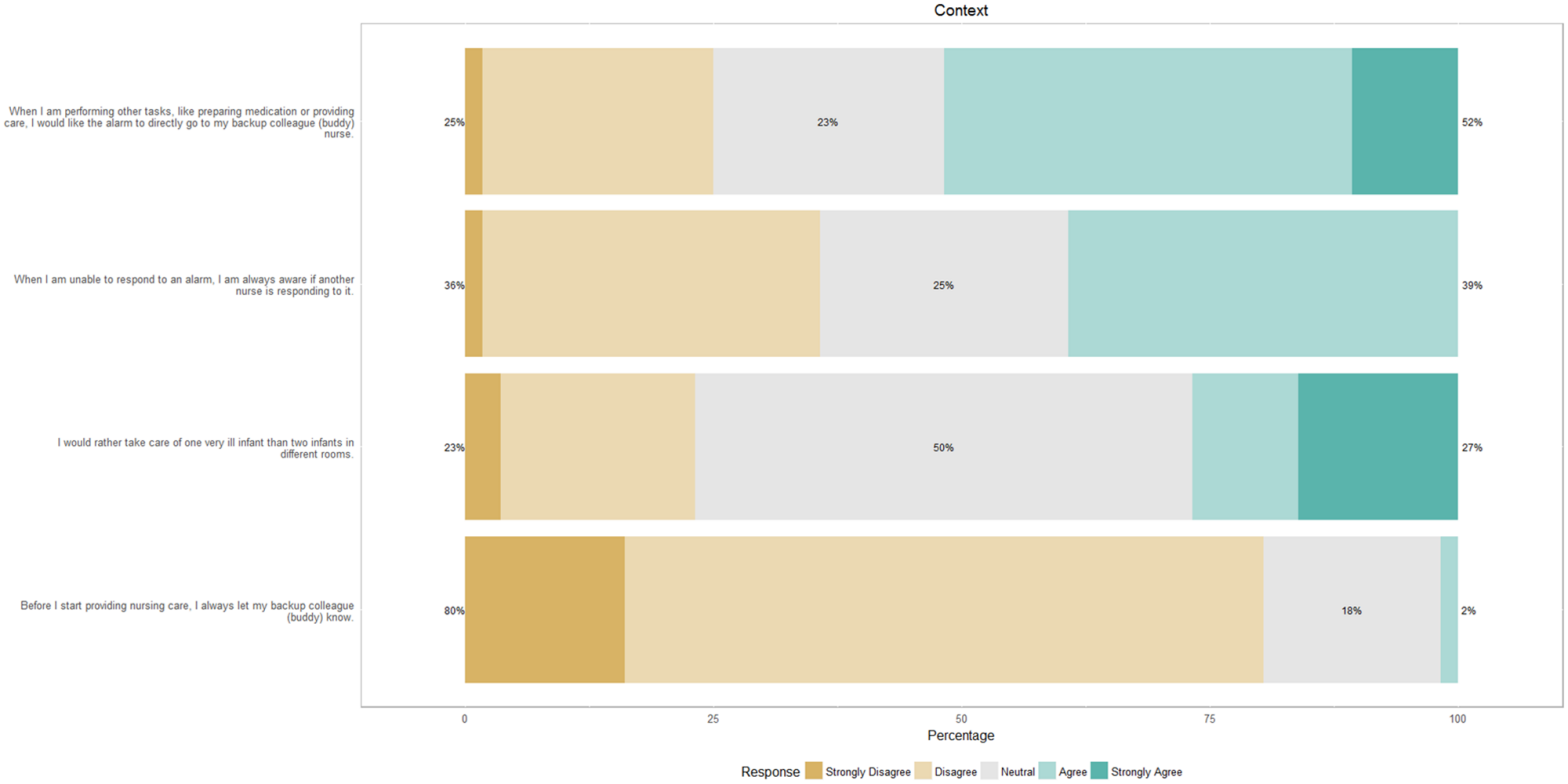
Response of nurses on the association between alarms and context.

Fig 6 shows the results about context, with 52% of the nurses stating that they would like the alarm to go to a buddy nurse when they are performing other tasks such as preparing medication or providing care. However, at present, they rarely let the buddy nurse know that they are going to start patient care.

During the video-monitoring study, a total of 5967 critical alarms were generated from ten infants in 2369 hours (99 days) of monitoring. The characteristics of these ten infants is listed in Table 1. 5451 out of the 5967 alarms (91% of total alarms) were recorded on video; the rest were missed because the Raspberry Pi was disconnected. The clinical condition, respiratory support and the nursing response to all critical alarms during video monitoring are shown in Table 2. Each infant provided a median of 250 (IQR, 230–271) hours of data during which time a median of 341 (IQR, 186–657) critical alarms were successfully recorded. For a median of 34% (IQR, 20–52) of these observed alarms, the nurse was already present in the room, and for a median of 13% (IQR, 10–16) of alarms, a parent was in the room. Nurses paused a median of 11% (IQR, 12–20) of all critical alarms. For alarms during the period the nurse was not in the room, the response rate within 90s was 26% with a median response time of 55s (IQR, 37-70s).

**Table 1:** Characteristics of the patient population enrolled in the video-monitoring study.

Feature	Median	25^th^ percentile	75^th^ percentile
Gestational age (weeks)	27	24.7	29
Birth weight (g)	982.5	800	1255
PMA, start of study (weeks)	29.3	27.6	31.7
PMA, end of study (weeks)	31.3	28.6	32.7
Length of stay (days)	33.5	24.5	55

**Table 2:** The clinical condition and respiratory support of ten preterm infants along with the nursing response to critical alarms based on video monitoring. The last row shows the median (IQR) values.

Clinical diagnosis during monitoring	Respiratory support during monitoring	Duration of monitoring (hours)	Alarms observed	Nurse in room (%)	Nurse response < 90s (%)	Alarms silenced (%)	Contribution of random effects to nurse responsiveness (odds ratio)
BPD, AoP	Whole period HFNC; room air	230	317	20	14	6	0.39
BPD, AoP	Whole period nasal CPAP with O2 therapy > 21%	279	1573	30	12	9	0.08
AoP	Whole period HFNC with O2 therapy > 21%	126	75	17	8	9	0.62
PDA treatment; AoP	Nasal CPAP 9 days; HFNC 3 days; room air	263	305	35	24	12	1.99
Uneventful course until final 12 hours: NEC with perforation	NCPAP 5 days and SIPPV 1 day with O2 therapy > 21%	135	172	56	18	15	2.8
Late-onset sepsis	All period nasal CPAP with O2 therapy > 21%	327	517	58	20	17	3.25
RDS, AoP	NCPAP 3 days; HFNC 8 days; Room air	239	186	33	23	9	1.75
IVH grade II, venous infarction; Late-onset sepsis	SIPPV 7 days; NCPAP 5 days; all period O2 therapy > 21%	271	657	52	18	16	2.66
AoP	Whole period HFNC with O2 therapy > 21%	260	365	19	10	7	0.40
AoP	Whole period NCPAP with O2 therapy > 21%	239	1284	42	16	18	1.70
-	-	250 [230–271]	341 [89–96]	34 [20–52]	17 [12–20]	11 [9–16]	1.7 [0.4–2.7]

Legend: BPD, Bronchopulmonary dysplasia; AoP, Apnea of prematurity; PDA, Patent ductus arteriosus; NEC, Necrotizing enterocolitis; RDS, Respiratory Distress Syndrome; IVH, Intraventricular hemorrhage; HFNC, High flow nasal cannula; NCPAP, Nasal continuous positive airway pressure; SIPPV, Synchronized Intermittent Positive Pressure Ventilation

Desaturation, bradycardia, physiological ventilator alarms (high peak inspiratory pressure alarm, disconnection of the ventilator circuit and problems with the endotracheal tube), apnea, asystole and ventricular tachycardia alarms comprised 99.7% of all observed critical alarms that occurred when the nurse was not in the room. The number of these alarms along with their duration and nurse response times are listed in Table 3. While desaturation, bradycardia and physiological ventilator alarms comprise 58.1%, 26% and 11.7% of all critical alarms when the nurse was not in the room, this changed to 47.6%, 14.6%, and 29% respectively when the nurse was present in the patient’s room. This is unsurprising since nursing care is associated with an increase in alarms associated with the ventilator and arterial blood pressure and this in turn changes the percentage contribution of the remaining alarms to the whole [5,10]. Additionally, nurses pause 23% of the alarms that are generated when they are in the room versus 7% of the alarms when they are not.

**Table 3:** The categories of critical alarms along with their prevalence, duration of alarm and the response time of nurses (if < 90s) for those alarms that were generated when the nurse was not in the patient’s room.

Alarm Category	Number of alarms (% of total alarms)	Duration (s), median (IQR)	Response time (s), median (IQR)
Desaturation	1978 (58.1)	10 (4–22)	56 (38–71)
Bradycardia	887 (26.0)	6 (3–16)	54 (41–68.75)
Physiological ventilator	397 (11.7)	9 (3–15)	51 (25–69)
Apnea	70 (2.1)	9 (5–18)	50.5 (40–69)
Asystole	31 (0.9)	4 (2–5)	41 (16.5–65.5)
Ventricular tachycardia	30 (0.9)	18 (7–18)	39 (30.75–52)

The regression model was significant with fixed effects alone (P-value <0.001) which shows that the response to alarm depends on the characteristics of the patient and the alarm. However, the addition of random effects, which account for dependencies in the data, improved the fit of the model to the data. After including random effects, the category of alarm and the duration of alarm were statistically significant. The odds of responding to a bradycardia alarm, compared to a desaturation alarm, were 1.47 (95% CI = 1.21–1.78; P-value <0.001) while that of responding to a ventilator alarm were 0.35 (95% CI = 0.27–0.46; P-value <0.001). For every 20s increase in the duration of an alarm, the odds of responding to the alarm within 90s increased to 1.15 (95% CI = 1.1–1.2; P-value <0.001).

The random effect corresponding to infants captured those infant-specific effects that were not explained by the fixed effects. The random effect explained 30% of the variance in response times. Typically, the odds of a response reduced in infants who had chronic illnesses such as BPD while it increased for infants that had sepsis/IVH/NEC (see Table 2).

## Discussion

To the best of our knowledge, this is the first study cataloging the nursing response to critical alarms in a private room intensive care setting along with an objective, video-annotated measure of nurse responsiveness to critical alarms. In a private room NICU context, nurses often have no line of sight to the patient and depend on technological means such as the central monitor, inter-bed communication, and handheld devices for receiving notifications of critical alarms. Upon receiving these alarms, they immediately look at their handhelds, seek waveform information from inter-bed communication or central posts and use background information of the infant in their decision to respond to an alarm. Only 23% of the nurses claim to immediately go to the patient’s room when notified of a critical alarm, despite the fact that the majority of nurses perceive most critical alarms as being clinically relevant. This implies that they value the insights and awareness of critical alarms being generated, even though the alarms may not necessitate clinical action. These results suggest that in light of the private room context, attempts to suppress or eliminate non-actionable clinical alarms should be supplemented by providing information on the physiological stability of infants so that nurses are not blind to acute changes in physiological status.

The median time to respond to the different types of critical alarms is long and varies between 39s for tachycardia to 54s and 56s for bradycardia and desaturation respectively. These response times are measured only in those cases where there is a response within 90s and therefore only when the nurse considered the alarm to be relevant enough to mandate a manual inspection by physically going to the patient’s room. This definition of clinical relevance is different from others used in literature such as that of Siebig et al. where an alarm is clinically relevant only if it was due to a technical problem or led to a diagnostic or therapeutic decision [17]. However, we consider such a definition to be more a measure of actionability than of relevance.

Based on the survey, nurses find bradycardia alarms to be the most reliable followed by desaturation and apnea alarms. This is also seen from the regression model where the odds of responding to a critical bradycardia alarm are significantly higher than that of responding to a desaturation alarm. Additionally, the odds of responding to alarms increases with the duration of the alarm. This result is similar to previous findings within a NICU context where the probability of acting on an alarm increased with the duration of the alarm [2].

Since the odds of responding to critical ventilator alarms are considerably lower than of responding to desaturation alarms, it appears that nurses attribute limited clinical relevance to ventilator alarms unless accompanied by monitor alarms. Although research on ventilator alarms is very limited, a previous study, although in an adult ICU setting, also found evidence of nurses attributing lower clinical relevance to ventilator alarms [5].

The random effects corresponding to infants explains 30% of the variation in responsiveness towards alarms. Markedly, nurse responsiveness is lower for infants with a chronic disease (BPD) in contrast with infants who have an acute burden of diseases such as sepsis, NEC or IVH. This suggests that nurses premeditate and prioritize responses to more vulnerable infants and have delayed or no response to alarms that are largely non-actionable, such as desaturation alarms from BPD infants. Overall, according to video monitoring, nurses responded to 26% of critical alarms in a median time of 55s, which is in agreement with the results of the questionnaire. The layout of the NICU does not appear to play a major role here since nurses do not believe that they are slow in responding to alarms and are non-equivocal about the distance that needs to be covered to respond to alarms. Although, it is likely that responses in bay area NICU are faster since there is no walking time.

An area of concern is that nurses perceive their response to critical alarms as slower when they are engaged in patient care or involved in other tasks such as preparing medications. This reduced ability to respond to critical alarms is also reported to be stressful, especially since nurses are not aware whether their buddy nurse is responding to the alarm. They agree that there should be a system to transfer all alarms to the buddy nurse in such situations. Since the survey suggests that nurses seek waveform information upon being notified of alarms, additional screens showing these waveforms should be set up in the NICU-pharmacy and dead spaces in the corridors. Furthermore, better coordination between buddy nurses might, to some extent, mitigate alarm fatigue.

Regarding yellow alarms, nurses report that they are neither stressed by them nor do they respond to them. However, they do find yellow alarms to be useful and want them to remain in the alarm chain. These findings do suggest that yellow alarms contribute to the heuristics employed by nurses in responding to critical alarms, so an option might be to change yellow alarms to just a visual notification and decrease unnecessary noise in the NICU. Overall, nurses do not feel that they receive too many alarms during the shift, are non-equivocal about the private room design reducing alarm fatigue and are unaffected by the geographical spread of the infants they have to care for. They do, however, find it more challenging to respond to alarms in a private room context. The busyness of the NICU with regard to the occupancy rate and the number of nurses working the shift does not appear to affect stress. Herein, the private room design might play a role since it censors noise from irrelevant alarms such as yellow alarms and alarms originating from infants that a nurse is not responsible for.

Nurses believe that infants generate more alarms when receiving care. This result is supported by the video monitoring data as well where nurses are present in the patient’s room for 35% of all critical alarms. Likely, a significant number of these alarms originate due to nursing care. Similar findings have also been reported earlier, albeit in an adult ICU population [17,18]. With an increasing drive towards private room NICUs and increase in familial involvement, parental presence is likely to increase. Luckily, nurses do not find this stressful, but a significant number do report faster response times to alarms when parents are in the room. This finding is opposite to that in a children’s hospital where parental absence improved nursing response to alarms [19].

The use of video monitoring for studying alarms is a powerful tool that can aid in determining the clinical relevance of alarms and has been used in several studies [11,17,20,21]. However, it is also known to be an expensive and time-consuming technology, with one study indicating that it costs more than US $300 to acquire and analyze each hour of video [22]. One of the strengths of this study is that it used low-cost computers for video monitoring that were programmed to take pictures every 5s, thereby reducing the data and the time required for manual inspection. Another strength of this study is the large number of alarms that were analyzed from different patients over a relatively long duration of time.

The limitations of the study include the fact that it is of a single-center design that employs an experienced group of nurses. Furthermore, the nursing response might have changed because nurses were aware of the recording, although the small and inconspicuous nature of the camera makes this unlikely. Finally, the regression model includes only a limited number of factors and the addition of other factors such as nursing experience, NICU occupancy, etc. can give more insights but at the expense of a proportional increase in the data needed for analysis.

## Conclusions

We surveyed nurses across broad themes related to alarms and contrasted these findings with those from a video-observation study to quantify nurse responsiveness to critical alarms. Nurses respond to only 26% of critical alarms in a median time of 55s. Using a regression model, we identified patient and alarm-level factors that increased the odds of a nurse responding to an alarm within the 90s. The clinical status of the patient, the category, and duration of the alarm modulate the responsiveness of nurses.

## Supporting information

S1 Document Questionnaire in English. (DOCX)

S2 Document Questionnaire in Dutch. (DOCX)
