# Supplementary material for: The heuristics of nurse responsiveness to critical patient monitor and ventilator alarms in a private room neonatal intensive care unit
Source: PLoS One. 2017 Oct 5;12(10):e0184567. doi: 10.1371/journal.pone.0184567 (PMC5628801; doi:10.1371/journal.pone.0184567)
Supplement: S1 Document — (DOCX) [file pone.0184567.s001.docx]

**S1 Appendix: Questionnaire in English**

1. How many years of experience do you have working as a NICU nurse?
2. What percentage of the time are you employed?
3. When a red alarm is generated on the handheld, I look at it immediately.
4. When a red alarm is generated, I immediately go to the patient’s room.
5. I have enough background information about an infant to decide whether or not to respond to a red alarm.
6. I always have the possibility to see the monitor waveforms for the patients I am responsible for.
7. For the patients I am responsible, I would like to have the possibility to see the monitor waveforms on the handheld.
8. When I am performing other tasks, like preparing medication or providing care, it is hard to react to a red alarm originating from another infant.
9. When I am engaged in patient care, I take more time to respond to a red alarm originating from another infant.
10. I would rather take care of one very ill infant than two infants in different rooms.
11. Infants generate more alarms when I am providing nursing care (diapering/suctioning etc.).
12. I always silence alarms (3 min) before starting nursing care (diapering/suctioning etc.).
13. If an alarm is generated in another room, I often use inter-bed communication on the patient monitor to look at parameters/alarms of that infant.
14. When an alarm is generated, I often use the central post to look at the parameters/alarms of that infant.
15. Before I start providing nursing care, I always let my backup colleague (buddy) know.
16. When I am performing other tasks, like preparing medication or providing care, I would like the alarm to go directly to my backup colleague (buddy) nurse.
17. My perception of alarm fatigue is dependent on the number of infants in the unit.
18. My perception of alarm fatigue is dependent on the number of nurses working in that shift.
19. The number of infants that I am responsible for changes during the shift.
20. During a shift, for short periods of time (e.g. between 5-60 minutes), the number of infants that I have to watch over (e.g. respond to alarms) can increase.
21. I often have the feeling that I am responsible for more infants than I can handle.
22. I often have the feeling that I have to watch over more infants than I can handle.
23. When an infant generates multiple red alarms in a short duration of time (e.g., 15 mins), my reaction time to the alarm decreases.
24. When I am unable to respond to a red alarm, I feel stressed.
25. I think that I am often slow in responding to red alarms.
26. I find red alarms stressful.
27. More than 90% of the red alarms I receive are clinically irrelevant and non-actionable.
28. I find red bradycardia alarms reliable.
29. I find red desaturation alarms reliable.
30. I find red apnoea alarms reliable.
31. I find yellow alarms useful.
32. I always respond to yellow alarms.
33. More than 90% of the yellow alarms I receive are irrelevant and non-actionable.
34. I find yellow alarms reliable, i.e. they are not artifacts.
35. I find yellow alarms stressful.
36. Yellow alarms are unnecessary and can be removed from the alarm chain.
37. I find parental presence in an infant’s room stressful.
38. I respond faster to red alarms if I know that parents are in the room.
39. I respond faster to yellow alarms if I know that parents are in the room.
40. Often, I have to cover large distances to respond to an alarm.
41. I experience more alarm fatigue when the infants I have to care for are widely spread in the unit.
42. My perception of alarm fatigue is lower because of the single room design.
43. The number of alarms that I get during my shift is not too many to handle.
44. When I am unable to respond to an alarm, I am always aware if another nurse is responding to it.
45. It is more challenging to respond to alarms in the single room NICU than in an open bay area NICU.
46. Alarms frequently interfere/interrupt my other nursing duties.
47. I regularly change alarm limits of infants on the patient monitor and the ventilator.
48. I always know which equipment (e.g. monitor, ventilator, and infusion pump) is responsible for generating an alarm.
49. The medical devices used in my unit (excluding the handheld) have distinct outputs (e.g., sounds, repetition rates, etc.) that allow me to identify the source of the alarm audibly.
50. I think I havent had sufficient training in properly using the patient monitor (e.g., data exploration, choosing ECG leads, alarm settings, usability).
