# Supplementary material for: The heuristics of nurse responsiveness to critical patient monitor and ventilator alarms in a private room neonatal intensive care unit
Source: PLoS One. 2017 Oct 5;12(10):e0184567. doi: 10.1371/journal.pone.0184567 (PMC5628801; doi:10.1371/journal.pone.0184567)
Supplement: S2 Document — (DOCX) [file pone.0184567.s002.docx]

**S2 Appendix: Questionnaire in Dutch**

1. Aantal jaren ervaring als NICU verpleegkundige.
2. Hoeveel procent is je dienstverband?
3. Wanneer een rood alarmgeluid klinkt op mijn pieper, kijk ik meteen op mijn pieper.
4. Wanneer een rood alarmgeluid klinkt, ga ik meteen naar de desbetreffende patiëntenkamer
5. Ik beschik over voldoende achtergrond informatie over de pasgeborene om te kunnen beslissen of ik op het rode alarm moet reageren of niet.
6. Ik kan altijd overal de curves van de bewakingsmonitor zien van de pasgeborenen waar ik verantwoordelijk voor ben.
7. Ik zou graag de mogelijkheid hebben om de curves van de bewakingsmonitor van de pasgeborenen waar ik verantwoordelijk voor ben, te kunnen zien op de telefoon/pieper
8. Wanneer ik met andere taken bezig ben, zoals het bereiden van medicatie, of het verzorgen van een pasgeborene, is het moeilijk om op een rood alarm te reageren wat van een andere pasgeborene komt.
9. Wanneer ik bezig ben met patiëntenzorg, duurt het langer om op een rood alarm van een andere pasgeborene te reageren.
10. Ik zorg liever voor één hele zieke pasgeborene op één kamer dan voor twee pasgeborenen in verschillende kamers.
11. Pasgeborenen genereren meer alarmen wanneer ik ze aan het verzorgen ben.
12. Ik onderdruk altijd het alarm (3 min) voordat ik start met de verzorging.
13. Wanneer een alarm afgaat in een andere kamer, gebruik ik de interbed communicatie op de bewakingsmonitor om naar de parameters/alarmen van die pasgeborene te kijken.
14. Wanneer een alarm afgaat, gebruik ik de centraalpost om naar de parameters/alarmen van die pasgeborene te kijken.
15. Vóórdat ik een kind ga verzorgen, laat ik dat altijd aan mijn achterwacht (buddy) weten.
16. Wanneer ik met andere taken bezig ben, zoals het bereiden van medicatie, het verzorgen van een pasgeborene, vind ik het fijn als mijn alarmen direct naar mijn achterwacht (buddy) gestuurd zouden kunnen worden.
17. Tijdens mijn dienst hangt het van het aantal pasgeborenen in de unit af hoe ik alarmen ervaar.
18. Tijdens mijn dienst hangt het van het aantal aanwezige verpleegkundigen in de unit af hoe ik alarmen ervaar.
19. Het aantal pasgeborenen waar ik voor moet zorgen verandert gedurende de dienst.
20. Als ik tijdens mijn dienst voor korte tijdperioden (Bijv. tussen5 – 60 minuten) 1 of 2 kinderen extra moet waarnemen, neemt het aantal alarmen toe voor mij.
21. Ik heb het gevoel dat ik verantwoordelijk ben voor meer pasgeborenen dan ik eigenlijk aan kan.
22. Ik heb het gevoel dat ik op meer pasgeborenen moet letten dan ik eigenlijk aan kan.
23. Wanneer een pasgeborene meerdere rode alarmen genereert in een korte tijdsperiode (bijv. 15 minuten), neemt mijn reactiesnelheid op het alarm af.
24. Ik voel me gestrest wanneer ik niet in staat ben om op een rood alarm te reageren.
25. Ik denk dat ik vaak langzaam reageer op een rood alarm.
26. Ik ervaar rode alarmen als stressvol.
27. Meer dan 90% van de rode alarmen die ik krijg zijn klinisch niet relevant/belangrijk en hoef ik geen actie op te ondernemen.
28. Ik vind rode bradycardiealarmen betrouwbaar.
29. Ik vind rode de-saturatie alarmen betrouwbaar.
30. Ik vind rode apneu alarmen betrouwbaar.
31. Ik vind gele alarmen nuttige informatie verstrekken over de conditie van de pasgeborene.
32. Ik reageer altijd op gele alarmen.
33. Meer dan 90% van de gele alarmen die ik krijg zijn klinisch niet relevant/belangrijk en hoef ik geen actie op te ondernemen.
34. Ik acht gele alarmen betrouwbaar, het zijn geen artefacten/storingen.
35. Ik ervaar gele alarmen als stressvol.
36. Gele alarmen zijn onnodig en kunnen verwijderd worden uit de alarmeringsketen.
37. Ik ervaar de aanwezigheid van ouders in de patiëntenkamer als stressvol.
38. Ik reageer sneller op een rood alarm als ik weet dat er ouders in de kamer aanwezig zijn
39. Ik reageer sneller op een geel alarm als ik weet dat er ouders in de kamer aanwezig zijn
40. Ik moet vaak een grote afstand afleggen om op een alarm te kunnen reageren.
41. Ik ervaar meer alarmvermoeidheid* als de kamers van de pasgeborenen waar ik voor moet zorgen verder uit elkaar liggen.

***** Met alarmvermoeidheid wordt bedoeld dat je door overmatig aantal alarmen minder goed of niet meer reageert op een alarm.

1. Ik ervaar minder alarm vermoeidheid door het ontwerp van de afdeling met één- persoonskamers.
2. Het aantal alarmen dat ik per dienst krijg is niet te veel om af te handelen.
3. Wanneer ik zelf niet in staat ben om op een alarm te reageren, dan weet ik altijd of een andere verpleegkundige reageert.
4. Het is een grotere uitdaging om op alarmen te reageren in een NICU met één persoonskamers dan op een NICU met zalen.
5. Het komt vaak voor dat alarmen mijn verpleegkundige taken verstoren.
6. Ik verander regelmatig alarmgrenzen van pasgeborenen aan de bewakingsmonitor of beademingsmachine.
7. Ik weet altijd wélk apparaat (bijv. bewakingsmonitor, beademingsmachine, infuuspompen) een alarm veroorzaakt.
8. De medische apparatuur in de unit (uitgezonderd telefoon/handheld) heeft verschillende

uitgangen (bijv. ander geluid, ander tempo van herhalingen), zodat ik in staat ben aan het geluid van het alarm te horen wat de bron is.

1. Ik vind dat ik onvoldoende training heb gehad om de bewakingsmonitor op de goede manier te kunnen gebruiken. (Bijv. het terugkijken van data, juiste afleidingen kiezen,

alarminstellingen, gebruiksgemak).
